# Unintended CRISPR-Cas9 editing outcomes: a review of the detection and prevalence of structural variants generated by gene-editing in human cells

**DOI:** 10.1007/s00439-023-02561-1

**Published:** 2023-04-24

**Authors:** John Murray Topp Hunt, Christopher Allan Samson, Alex du Rand, Hilary M. Sheppard

**Affiliations:** School of Biological Sciences, University of Auckland, Auckland, New Zealand

## Abstract

Genome editing using the clustered regularly interspaced short palindromic repeats (CRISPR) and CRISPR-associated protein (Cas) gene-editing system (CRISPR-Cas) is a valuable tool for fundamental and applied research applications. Significant improvements in editing efficacy have advanced genome editing strategies into phase 3 human clinical trials. However, recent studies suggest that our understanding of editing outcomes has lagged behind the developments made in generating the edits themselves. While many researchers have analyzed on- and off-target events through the lens of small insertions or deletions at predicted sites, screens for larger structural variants (SVs) and chromosomal abnormalities are not routinely performed. Full and comprehensive validation of on- and off-target effects is required to ensure reproducibility and to accurately assess the safety of future editing applications. Here we review SVs associated with CRISPR-editing in cells of human origin and highlight the methods used to detect and avoid them.

## Introduction

The development of the clustered regularly interspaced short palindromic repeats (CRISPR) and CRISPR-associated (Cas) protein gene-editing system (CRISPR-Cas) in 2012 transformed our ability to treat genetic diseases by enabling targeted-modification of intracellular DNA. Monogenic diseases are the most common target of CRISPR gene therapy, and some, such as sickle cell disease or transfusion-dependent beta-thalassemia (TDT), have recently advanced into phase 3 clinical trials (clinical trial.gov: NCT03655678/NCT03745287/NCT05477563). However, the CRISPR-Cas system is not limited to simple hereditary diseases, and human clinical trials have begun for the treatment of other conditions, such as cancers (clinical-trial.gov: NCT04976218) or bacterial (clinical-trial.gov: NCT04191148) and viral infections (clinical-trial.gov: NCT05144386).

### Gene editing using CRISPR-Cas9

The CRISPR-Cas system excels due to its capacity to provide inexpensive, accessible, and robust editing without a requirement for retroviral integration. Using this approach, gene-editing is typically facilitated by the CRISPR-associated protein 9 (Cas9), an endonuclease which cleaves double-stranded DNA at genomic loci defined by a 20-nucleotide guide RNA (gRNA) and a three-nucleotide protospacer adjacent motif (PAM) (Doudna and Charpentier 2014). In humans, double-stranded DNA breaks (DSBs) are primarily repaired by the error-prone non-homologous end joining (NHEJ) pathway, typically resulting in small (up to ten nucleotide) insertion and deletion events (INDELs) (Allen et al. 2018; Chang et al. 2017). INDELS can be targeted to promoter or protein-coding regions to directly disrupt gene activity and can be used to ablate gene expression. Specific DNA changes can be achieved by providing an appropriate DNA template to employ the endogenous homology repair or single-strand template repair pathways in a process referred to as homology directed repair (HDR) (Lee et al. 2022). Genome editing by HDR is the mainstay of personalized gene therapy as it can be used to precisely correct patient-specific disease-causing mutations or to generate genotypes of interest for disease modeling.

### Gene editing can lead to the unintended generation of structural variants

**Fig. 1:**
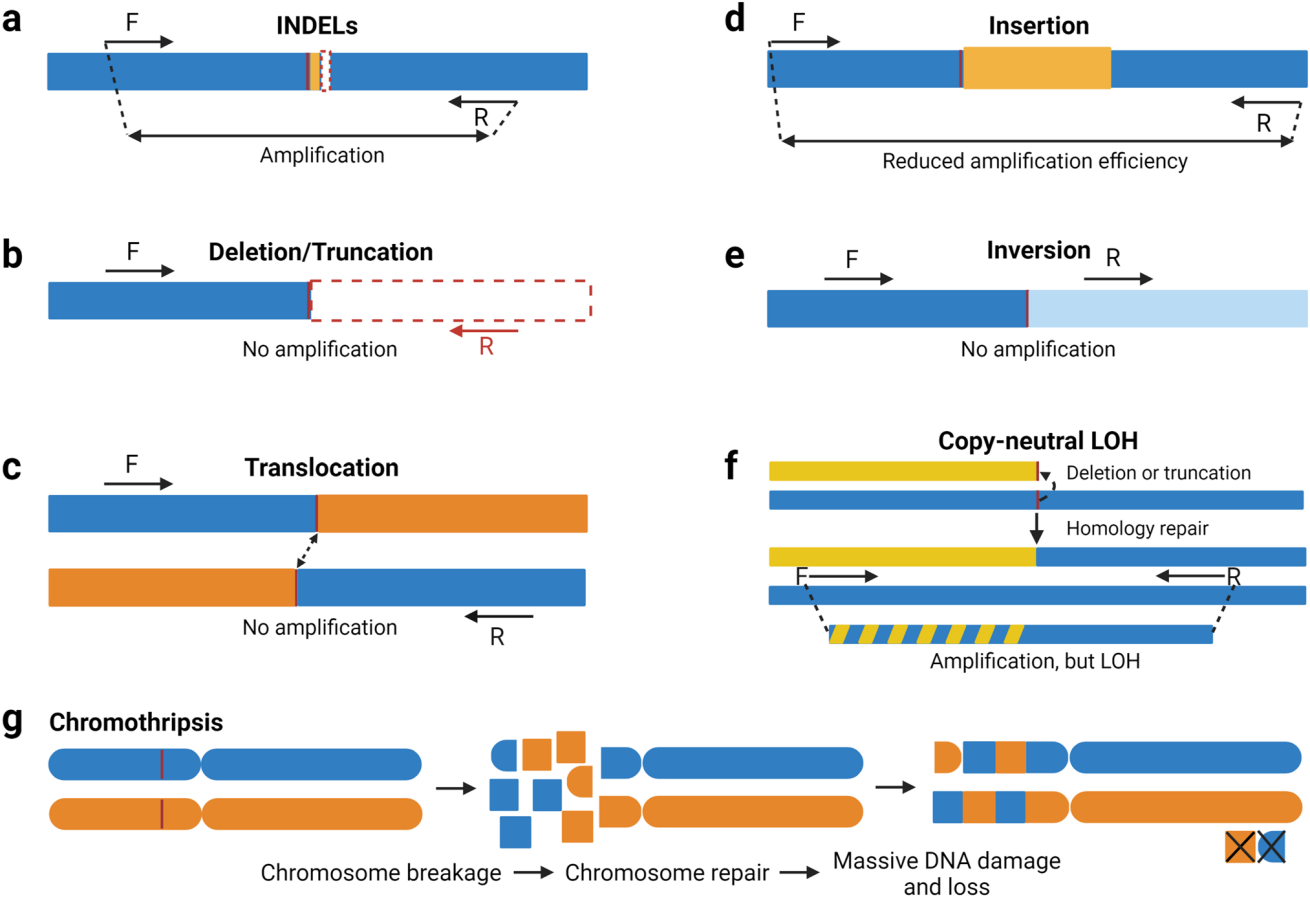
Schematic representation of possible editing outcomes which may be induced by CRISPR-Cas9 genome editing. Standard alleles are represented by blue bars, with the CRISPR-Cas cleavage site indicated by the red vertical line. **a**–**f** INDELs and SVs classes are depicted with standard genotyping PCR primers (forward (F) and reverse (R)) to indicate whether an amplicon would be produced after each editing outcome. **f** Copy-neutral loss of heterozygosity (CN-LOH) occurs when one chromosomal region is lost and then repaired by various homology-dependent mechanisms, leading to two identical copies of the genome downstream of the cleavage site. Yellow and blue colorings in **f** indicate homologous chromosomes. Hatched blue/yellow indicates that the F primer can bind to either of the homologous chromosomes. **g** Chromothripsis is a complex chromosomal rearrangement characterized by multiple simultaneous chromosome break and repair events leading to DNA rearrangements and loss

The primary concern when considering the clinical application of any gene therapy is the potential for unintended genome alterations that may create genomic instability or interfere with regular gene function. Accordingly, it is important to be aware that the CRISPR-Cas system may generate undesired, genotoxic side effects. For example, CRISPR-Cas gRNAs may tolerate small DNA mismatches and cause DNA cleavage and thus INDELs at off-target sites (Han et al. 2020). Furthermore, the improper repair of any DSBs, either at on- or off-target sites has the potential to induce larger genomic aberrations (> 50 bp) known as structural variants (SVs) (Mahmoud et al. 2019). These structural variants can be broadly categorized into deletions, duplications, insertions, inversions, translocations, viral vector integrations, and more complex events such as chromothripsis (Fig. 1) (Mahmoud et al. 2019). While exogenous DNA insertions, such as template insertions and viral vector integrations, comprise a distinctive group of SVs compared to endogenous DNA insertions, for the purposes of this review, we have chosen to classify these insertions as part of the general insertion SV group. Given that recent studies indicate that SVs can play a key role in driving tumorigenesis (Alhafidz and Ailith 2022), the generation of SVs, however rare, should be a key consideration for any gene therapy. It is worth acknowledging that the oncogenic potential of SVs is dependent on the specific SV and the permissibility of the cell to transformation (Dubois et al. 2022). Additionally, while the analysis of SVs resulting from CRISPR-editing is not well reported in clinical trials, there have been no reports of cancer incidence associated with CRISPR-based therapies to date.

Despite the rapid advancements made with the CRISPR-Cas gene-editing system, our understanding of editing outcomes has lagged behind the developments made in generating the edits themselves. Although the potential for CRISPR-Cas9 editing to cause translocations and large deletions was documented in 2014 (Choi and Meyerson 2014; Maddalo et al. 2014), it was not until four years later that the full extent of the generation of on-target SVs began to be unraveled (Kosicki et al. 2018). Subsequently, on-target, mega-base-scale deletions, insertions, chromosomal truncations, copy-neutral loss of heterozygosity (CN-LOH), translocations, and chromothripsis events have all been described as a result of CRISPR-Cas editing in human cells (Boutin et al. 2021, 2022; Cullot et al. 2019; Kosicki et al. 2018; Leibowitz et al. 2021; Liu et al. 2021; Rayner et al. 2019; Turchiano et al. 2021; Weisheit et al. 2020; Yin et al. 2019; Zhang et al. 2021). Still, to date, the prevalence of SVs in CRISPR-Cas edited human cells is largely unknown. This delay in understanding is likely due to the most widely used on- and off-target editing analysis methods being limited in their ability to detect SVs (see section "Evidence for CRISPR-associated SVs"). If SVs remain undetected, they may alter either the reported editing frequency, cell genotype, or skew assay results by altering cellular function, and thus they have the potential to corrupt entire studies (Blondal et al. 2021; Rayner et al. 2019; Weisheit et al. 2020). Furthermore, the rapidly advancing cell-based CRISPR therapeutic strategies can require millions to billions of edited cells to be engrafted to a patient (Boutin et al. 2021). It is conceivable that within a pool of edited cells, SVs may confer a growth advantage or even promote an oncogenic state. Thus, a comprehensive understanding of SV outcomes in CRISPR-edited cell populations may be prudent to ensure the safety of future gene therapy approaches. As CRISPR-Cas editing strategies are highly diverse, it is likely that the predominant types of SVs and their frequencies will need to be assessed for each unique application.

## Evidence for CRISPR-associated SVs

### SVs in human cancer cells lines

Genome-edited cancer cell lines are desirable for the study of cancer biology and therapeutics. However, many cancer-derived cell lines exhibit high levels of chromosomal instability, in part due to aberrant DNA repair mechanisms, such as inactivation of the tumor suppressor protein, p53 (Rayner et al. 2019). Thus, it is feasible that the induction of DSBs in these cancer cell lines may further compromise genome integrity and cause widespread complex SVs.

In the well-studied HEK293T cancer cell line, kilobase-sized deletions and inversions have been detected at frequencies of ~ 3% (0.1–5 kilobase (kb)) (Yin et al. 2019; Zhang et al. 2021), and 0.05% (5–50 kb), following the induction of a single DSB (Yin et al. 2019). Distal chromosome arm truncations have been detected at frequencies of 10–25.5% in edited HEK293T cell clones, independent of the target loci (Boutin et al. 2021; Cullot et al. 2019). Intra-chromosomal translocations have also been detected, making up to 6.2–14% of editing outcomes in one study utilizing HEK293T cells (Liu et al. 2021; Yin et al. 2019; Zhang et al. 2021). Interestingly, chromosomal translocations occurred at similar frequency between both predicted off-target sites and low-level genome-wide DSB events, which suggests that translocations may be possible even in the absence of off-target DSBs (Yin et al. 2019; Zhang et al. 2021).

Similarly, widespread chromosomal instability, including intra-chromosomal translocations and distal chromosome arm truncations as a result of CRISPR-editing has been described in well-defined colorectal cancer cell lines (Przewrocka et al. 2020; Rayner et al. 2019). CRISPR-associated chromosome instability was more prominent in cancer cell lines, which exhibit aneuploidy (COLO320 and SW1463) than those with a more stable karyotype (HCC2998 and HTC116). Przewrocka et al. (2020), identified chromosomal truncations in CRISPR-edited HCT116 cells at rates of 2–7%. RNA sequencing of the affected clones showed that the most significantly downregulated genes were those located on the truncated chromosome arm distal to the target site, highlighting a functional consequence of these SVs (human cell studies are summarised in Table 1).

**Table 1:** Summary of papers that have identified SVs in human cells

Cell category	Cell line(s) or type(s)	Genotoxicity type	References
Cancer cell lines	HEK293(T), K562, COLO320, SW1463, HCC2998, HTC116, HAP1, Hep2G	Chromosomal truncations, translocations, kilobase and megabase deletions, insertions, complex insertions and deletions	(Boutin et al. 2021; Cullot et al. 2019; Geng et al. 2022; Liu et al. 2021; Przewrocka et al. 2020; Rayner et al. 2019; Xin et al. 2022; Yin et al. 2019, 2022; Yoo et al. 2022; Zhang et al. 2021)
Primary cells	hTERT-fibroblasts, hTERT-RPE1	Kilobase deletions, insertions and rearrangements, chromosomal truncations, micro-nucleation and chromothripsis	(Cullot et al. 2019; Kosicki et al. 2018; Leibowitz et al. 2021)
	Fibroblasts	Kilobase and megabase deletions, complex insertions and deletions, micro-nucleation	(Cullot et al. 2019; Leibowitz et al. 2021)
	T-cells	Kilobase deletions, copy-neutral loss of heterozygosity, translocations	(Wen et al. 2021; Yin et al. 2022)
	iPSCs	Kilobase deletions, copy-neutral loss of heterozygosity, insertions	(Simkin et al. 2022; Weisheit et al. 2020; Wen et al. 2021)
	HSPCs	Kilobase deletions, insertions, copy-neutral loss of heterozygosity, translocations, micro-nucleation	(Boutin et al. 2021; Leibowitz et al. 2021; Turchiano et al. 2021; Wen et al. 2021)
Embryonic cells	ESCs	Kilobase and megabase deletions, chromosome loss, copy-neutral loss of heterozygosity	(Bi et al. 2020)
	Zygotes/Embryos	Kilobase deletions, truncations, copy-neutral loss of heterozygosity, chromosome loss	(Alanis-Lobato et al. 2021; Zuccaro et al. 2020)

### SVs in primary cells, immortalized primary cells, iPSCs and HSPCs

Primary cells and stem cells are useful for basic and translational research as they more closely reflect in vivo cells compared to cancer cell lines and they are the foundation of cell-based gene therapies. Kiosicki et al. (2018) reported extensive on-target SVs in single-DSB edited human cells (human telomerase reverse transcriptase (hTERT) immortalized retinal pigment epithelium cells (RPE1)), which included kilobase-sized deletions, insertions, and rearrangements (Kosicki et al. 2018). Subsequently, gene-editing related SVs have been reported in other primary and stem cells including hTERT-immortalized fibroblasts, induced pluripotent stem cells (iPSCs), and hemopoietic stem and progenitor cells (HSPCs) (Blondal et al. 2021; Boutin et al. 2021; Simkin et al. 2022; Turchiano et al. 2021; Weisheit et al. 2020; Wen et al. 2021).

Regarding induced pluripotent stem cells, one study indicated that up to 40% of iPSC clones had on-target SVs, including 0.5–4 kb deletions or CN-LOH of the entire chromosome arm distal to the target site (Weisheit et al. 2020). In this study, one of the target loci was the amyloid precursor protein (*APP*) encoding gene. Cortical neurons derived from iPSCs with SVs had APP expression reduced by ~ 50% compared to those without SVs. The authors noted that the reduction in APP expression may have significantly altered the disease modeling of the iPSC affected by SVs (Weisheit et al. 2020). Other recent studies also noted deletions over 100 bp (< 1.5%) (Wen et al. 2021), plasmid or mitochondrial DNA insertions, and CN-LOH as editing outcomes in iPSCs (Blondal et al. 2021; Simkin et al. 2022).

In hematopoietic stem and progenitor cells, kilobase-sized deletions or copy-neutral SVs seem to be the predominant on-target SVs and have been identified in up to 20% of edited cells (Boutin et al. 2021; Turchiano et al. 2021; Wen et al. 2021). An in-depth analysis of edited HSPCs by CAST-seq (a method described later in section "Methods to detect structural variants in CRISPR-edited cells") showed that large deletions, inversions, and chromosomal translocations with the homologous chromosome made up ~ 19.5% of total edited alleles (Turchiano et al. 2021). In comparison, inter-chromosomal translocations with off-target or genome-wide DSBs were only detected in approximately 0.5% of edited alleles. In a separate study, LOH of the target chromosome arm was detected at a frequency of ~ 1%, the majority being CN-LOH events (Boutin et al. 2021). Significantly, HSPC clones with CN-LOH exhibited abnormal methylation patterns and aberrant expression of two known tumor suppressor genes and one oncogene, again highlighting a previously underappreciated consequence of SVs (Boutin et al. 2021).

### SVs in human embryonic stem cells, zygotes, and embryos

CRISPR-Cas9 genome editing has been reported to induce SVs in both mouse embryonic stem cells (ESC) and mouse embryos (Adikusuma et al. 2018; Kosicki et al. 2018; Owens et al. 2019). Recent studies suggest that this may also occur in genome edited human ESC and embryos (Alanis-Lobato et al. 2021; Bi et al. 2020; Zuccaro et al. 2020). In one study of CRISPR-edited human embryonic stem cells, SVs comprised up to 5.4% of detected editing events (Bi et al. 2020). Of these, 78–98% were deletions ranging from 31 to 5500 bp, although insertions and inversions were also detected. However, due to the limitations of the detection methods used in this study (IDM-seq and SNP genotyping; see section "Methods to detect structural variants in CRISPR-edited cells"), the frequency of chromosomal aberrations and CN-LOH in hESCs remains unknown. Recently, two studies in human embryos demonstrated segmental and whole chromosome losses from induced single DSBs (Alanis-Lobato et al. 2021; Zuccaro et al. 2020). Zuccaro et al. (2020) used SNP genotyping and qPCR to demonstrate that LOH of SNP sites was due to a loss of DNA from chromosome arm truncations, as opposed to homology directed repair that was reported in a previous study (Ma et al. 2017). Similarly, Alanis-Lobato et al. (2021) demonstrated that segmental deletions of 4 kb to at least 20 kb occurred in 16% of cells from embryos that were edited with a single DSB (Alanis-Lobato et al. 2021). A recent study in CRISPR-edited macaque embryos also identified large on-target deletions ranging from ~ 0.2 kb to ~ 5 kb, inversions, duplication, and *de novo* mutations at off-target sites (Schmidt et al. 2023).

### HDR-enhancing techniques may increase the incidence of structural variants in CRISPR-edited cells

In general, gene-editing by HDR is currently limited by low editing efficacy and high variability of editing efficacy between loci and cell type, despite high on-target cutting efficiencies (Lee et al. 2022). As a result, positive selection or clonal isolation of edited cells may be required to attain experimentally useful HDR levels, which is not feasible for all editing applications. This has led to the development of HDR-enhancing techniques such as cell cycle synchronization or the chemical modulation of repair pathways (Lee et al. 2022). For example, the transient inhibition of the tumor suppressor protein, p53, has been reported to increase the efficiency of CRISPR-mediated HDR by up to 17-fold in hPSCs (Ihry et al. 2018), making it a desirable target for knockdown or inhibition (Schiroli et al. 2019). However, as p53 is a key regulator of DNA repair and growth arrest, a concern is that the induction of DSBs in p53-deficient cells may, in turn, increase the mutational burden and SV incidence in the edited cells (Mirgayazova et al. 2020). This has been explored in hTERT-immortalized fibroblasts (hTERT-fibs), where a significant increase in CRISPR-induced chromosomal truncations was reported in p53-inactivated hTERT-fibroblasts (7.7%) compared to their p53 intact counterparts (1.1%) (Cullot et al. 2019). Furthermore, in p53-depleted hTERT-RPE1 cells, those that had micro-nucleation post-editing led to granddaughter cells with chromothripsis in 72% of cases (Leibowitz et al. 2021). Although micro-nucleation was also observed in p53-competent cells at rates of up to 2.5% in hSPCs, 3% in primary foreskin fibroblasts and 7.5% in hTERT-RPE1 cells, the affected cells were ~ 50% less likely to undergo cellular division at the first cell cycle post editing (Leibowitz et al. 2021). Furthermore, in p53-active human hematopoietic stem cells, the percentage of alleles containing unbalanced rearrangements and translocations reduced over several cell cycles, likely due to negative selection pressure of these mutations (Turchiano et al. 2021). In light of this, further research is required to identify if temporary p53 inactivation can increase HDR efficiency, without increasing the incidence of long-term viable SVs in an edited population of cells. Furthermore, these data suggest that rigorous analysis for chromosomal aberrations should be standard when editing cell lines with reduced p53 capacity.

NHEJ-inhibitors (e.g., M3814 or NU7441), which inhibit key NHEJ repair pathway proteins, such as KU or DNA-PK4, have been shown to improve HDR-efficacy by four- to five-fold, independent of the target loci (Chu et al. 2015; Riesenberg et al. 2019). However, recently it has been shown that inhibition of NHEJ proteins results in increased incidence of large deletions, insertions, translations and chromosomal truncations as a result of CRISPR-Cas-mediated DNA cleavage (Do et al. 2012; Kosicki et al. 2022; Liu et al. 2021; Quan et al. 2022; Wen et al. 2021). Therefore, broad detection of editing outcomes, including on-target SVs, is essential for primary research and clinical therapies which incorporate the use of NHEJ-inhibitors.

### Alternative CRISPR strategies may reduce the incidence of SVs

The conventional CRISPR-Cas system relies on introducing potentially genotoxic DSBs using the Cas9 nuclease, which can result in extensive DNA damage if not repaired correctly. High-fidelity Cas9 nuclease variants can reduce off-target effects, such as INDELs and translocations (Yin et al. 2019); however, they may not be able to prevent translocations between low-level DSBs or isolated SVs at the target site (Turchiano et al. 2021; Yin et al. 2022; Zhang et al. 2021). Cas9-nickases are catalytically modified to induce single-stranded DNA breaks, which may reduce the rate of off-target edits and on-target SVs associated with DSBs. For example, a single-nicking strategy significantly decreased the frequency of chromosomal truncations compared to a standard DSB strategy in a study using HEK293T cells (undetected versus 10% respectively) (Cullot et al. 2019). Similarly, switching from a Cas9 nuclease to a double Cas9-nickase strategy reduced translocation frequency from 2.7 to 0.5% in HEK293T cells, albeit at the expense of an up to 50% loss in editing efficiency (Yin et al. 2019). Both the Cas9-nickase-derived base editors (BE) and prime editors (PE) can also improve editing precision (Anzalone et al. 2020). Base editors introduce specific point changes at targeted sites and have recently demonstrated high efficiency with low rates of INDELS and SVs (Liao et al. 2023; Yin et al. 2022). For example, in HEK293 cells, both a cytosine (BE4max) and adenine (ABEmax) BE generated translocations at a frequency of 0.22% and 0.19% respectively compared to 1.93% with a regular Cas9 nuclease (Yin et al. 2022).

The Cas9TX variant, which fuses Cas9 nuclease with a 3′–5′ exonuclease can also reduce translocations by promoting end processing and thus decreasing re-cutting (Yin, Fang, et al., 2022; Yin et al. 2022). This was demonstrated in HEK293T cells where Cas9TX had a reduced translocation frequency of 0.42%, compared to 1.93% of regular Cas9 nuclease (Yin et al. 2022). Cas12 is another type of CRISPR enzyme that is emerging as a promising alternative to Cas9 for genome editing (Xin et al. 2022). As shown in a recent study, Cas12 can reduce the occurrence of translocations, large deletions, and viral vector integrations when compared to regular Cas9 (Xin et al. 2022). In HEK293 cells, Cas12f variants were found to reduce translocations by two to threefold compared to Cas9 (with translocation rates of approximately 1.17% versus 3.55%, respectively), albeit with a generally lower editing efficiency (Xin et al. 2022). As novel CRISPR-editing tools continue to emerge, it is important to evaluate their effects on the occurrence of SVs, such as translocations, in addition to their editing efficiency. This would provide valuable insights into the overall performance and safety of these new systems.

### Retention of SVs post editing

One way to assess the impact of SVs is by tracking their prevalence over time (see section "Clonal expansion assays"). Numerous studies have found that the occurrence of SVs decreases after several cell cycles following editing, which may be due to inhibition in cell growth during cell cycle checkpoints (Boutin et al. 2021; Turchiano et al. 2021; Yin, Lu, et al. 2022). However, the persistence or clonal expansion of a particular edit, including SVs, could indicate a mutation that is either tolerated or potentially confers a positive selective growth advantage. For example, two months following the infusion of autologous edited T cells into mice, Wu et al. found that the translocation frequency in the cell population had reduced, but translocations still persisted (0.98% in *ex vivo* activated T cells versus 0.17% to 0.59% in T cells two months after transplantation) (Wu et al. 2022). In this case, the authors speculated that the retention of the remaining translocations was likely due to a passenger effect from general expansion of the T cells rather than translocation-driven selection. Nonetheless, despite a trend toward decreasing numbers of SV carrying cells demonstrated in previous studies (Boutin et al. 2021; Turchiano et al. 2021; Yin et al. 2022), these studies indicate that SVs can persist for many months (Wu et al. 2022).

## Current methods used to analyze CRISPR edits

The most common CRISPR-edit analysis methods typically involve the generation of short amplicons (< 1 kb) by polymerase chain reaction (PCR) spanning the edited region of interest. Amplicons are then sequenced using either Sanger or next-generation sequencing (NGS). This short amplicon sequencing is favored as it is rapid, relatively inexpensive, and well supported with user-friendly bioinformatic tools for analysis (C Li et al. 2022). The key limitation of short-amplicon sequencing is that it can only detect mutations that are housed within the relatively small amplicon, which renders it unable to detect the majority of SVs (see Fig. 1). For example, in the case of a 1 kb amplicon, deletions (i.e. a unidirectional deletion of > 500 nucleotides), translocations, or other SVs may remove a primer amplification site(s) and prevent amplification and therefore detection. Additionally, insertions or duplicated regions may push primer annealing sites apart potentially reducing amplification efficiency with standard PCR cycle settings. Therefore, short amplicon analysis cannot confirm the absence of undesired edits as it cannot detect the vast majority of SVs.

One commonly used approach to identify off-target INDELs and certain types of SVs involves whole genome sequencing (WGS), specifically using short-read Illumina WGS (SR-WGS) (Li et al. 2019). Whole exome sequencing (WES) and total RNA sequencing (RNA-seq) are also used but have reduced scope compared to WGS as they only encompass the coding regions of the genome. A key limitation of SR-WGS is that the standard 30x genome coverage lacks the read depth to detect low-frequency mutations (INDELs or SVs) in pooled, heterozygous edited DNA. Increasing read depth would enhance the detection of rarer variants, but this comes with a significant increase in cost. Additionally, while currently available SV detection algorithms can detect high frequency SVs of all types, detection of SVs present at a frequency below 20% in pooled cell populations continues to be difficult to perform with adequate sensitivity, even at average sequencing depths exceeding 90x (Gong et al. 2021). An alternative solution is to analyze many individual clones isolated from the original mixed pool of edited cells. Although this would enable the detection of SVs (Alanis-Lobato et al. 2021; Schmidt et al. 2023; Simkin et al. 2022), this method can be expensive, labor-intensive, and would be difficult to achieve sufficient depth to identify rare variants, requiring the analysis of hundreds of cell clones, which makes it unsuitable for use in many settings.

## Methods to detect structural variants in CRISPR-edited cells

Currently, there is no single method which can comprehensively detect all SVs present in heterogeneous, pooled, edited DNA in an unbiased genome-wide manner. However, many techniques ranging from cytogenetic analysis to novel NGS-library preparations have been developed to detect and characterize editing-associated SVs (as summarized in Table 2).

**Table 2:** Methods used or developed to detect SVs in edited human cells

Method	Description	Advantages	Limitations	References
Fluorescent in situ hybridization (FISH)	Chromosome structure is analyzed using defined fluorescent DNA probes observed in condensed chromosomes	Can detect large chromosomal aberrations, such as truncations and translocations	Has low-resolution and sensitivity. Is time-consuming when scaled. Requires live cells	(Boutin et al. 2021; Cullot et al. 2019; Leibowitz et al. 2021; Przewrocka et al. 2020; Rayner et al. 2019)
Array-comparative genomic hybridization (aCGH)	Genome wide DNA copy number analysis is performed based on the relative intensity of complementary fluorescent probes between sample and control DNA	Can detect SVs that result in copy-number gain or loss	Detection threshold not suitable for pooled DNA. Cannot detect the location of copies or copy number neutral SVs	(Boutin et al. 2021; Cullot et al. 2019; Przewrocka et al. 2020)
Quantitative PCR (qPCR) and quantitative genotyping PCR (qgPCR)	A modified PCR which can quantify the allele copy number of a target site post editing	Can detect SVs that result in copy-number gain or loss of the specific amplification site	Detection threshold not suitable for pooled DNA. Cannot detect the location of copies or copy number neural SVs	(Boutin et al. 2021; Simkin et al. 2022; Weisheit et al. 2020, 2021)
Single-nucleotide polymorphism (SNP) genotyping	Heterozygous SNP allelic ratios are analyzed by either targeted amplification and sequencing or SNP-array methods	Can detect chromosomal aberrations or SVs which result in LOH of SNP sites e.g., deletions and truncations	Detection threshold not suitable for pooled DNA. Cannot distinguish between deletions and CN-LOH	(Alanis-Lobato et al. 2021; Leibowitz et al. 2021; Przewrocka et al. 2020; Simkin et al. 2022; Weisheit et al. 2020; Zuccaro et al. 2020)
Targeted short- and long-amplicon sequencing	Targeted PCR amplification followed by NGS	Can detect SVs housed completely within the amplicon	Limited to SVs housed completely within the amplicon. Allele quantification is prone to PCR and sequencing bias	(Kosicki et al. 2018; Quan et al. 2022; Simkin et al. 2022; Wen et al. 2021; Yoo et al. 2022)
Individual DNA molecule sequencing (IDMseq)	Individual DNA molecules are labeled with UMIs followed by the generation of short- or long amplicons and NGS	Detect and quantify SVs housed completely within the amplicon	Limited to SVs housed completely within the amplicon	(Bi et al. 2020)
Linear amplification-mediated high-throughput genome-wide translocation sequencing (LAM-HTGTS)	Linear amplification across a target DSB site using a biotinylated primer. Amplicons are enriched by streptavidin selection and are further amplified after the ligation of an adapter. Amplicons not containing SVs are ablated using a rare-cutting restriction enzyme and the remaining amplicons are sequenced by NGS	Can detect SVs where the break point is proximal to the primer site	Cannot detect SVs where the primer site has been ablated. Allele quantification is prone to PCR and sequencing bias	(Hu et al. 2016)
Primer extension-mediated sequencing (PEM-seq)	Primer extension across a target DSB site using a biotinylated primer. Amplicons are enriched by streptavidin followed by the ligation of a barcoded adapter. Nested amplification is performed before sequencing by NGS	Can detect and quantify SVs where the break point is proximal to the primer site	Cannot detect SVs where the target primer site has been ablated	(Liu et al. 2021; Wu et al. 2022; Xin et al. 2022; Yin et al. 2019; Yin et al. 2022; Zhang et al. 2021)
Chromosomal aberrations analysis by single targeted linker-mediated PCR sequencing (CAST-seq)	Pooled DNA is fragmented and linkers are ligated. Fragments are then amplified by PCR using ‘bait’ and linker primers so that fragments which do not contain SVs are ablated by the included ‘decoy primers’. The amplicons are then sequenced by NGS	Can detect SVs where the break point is proximal to the primer site	Cannot detect SVs where the target primer site has been ablated. Quantification requires ddPCR calibration. Does not quantify DNA without SVs	(Turchiano et al. 2021)
Whole-genome sequencing	Unbiased next-generation sequencing of whole genomic DNA	Can detect all types of variants including SNPs, INDELs and SVs at all sites	Detection threshold not suitable for pooled DNA	(Alanis-Lobato et al. 2021; Schmidt et al. 2023; Simkin et al. 2022)
Xdrop	Encapsulated PCR of fragmented HMW DNA with primers for a 100–200 bp target, distal (< 5 kb) to the break point. Droplets containing the fragments of interest can be identified with an intercalating fluorescent dye and sorted by flow cytometer droplet sorting. Enriched HMW DNA is then amplified by MDA followed by NGS	Can detect SVs where the break point is within the enriched HMW DNA	Cannot detect SVs where the target primer site(s) has been ablated	(Blondal et al. 2021; Geng et al. 2022)
Prime editor-assisted off-target characterization (PEAC-seq)	A Cas9, M-MLV reverse transcriptase fusion protein is used to generate and tag DSBs at off-target sites. Bulk DNA is fragmented by Tn5 tagmentation, which introduces UMI and adapter sequences. For identification of translocations, nested PCR is performed with target-specific forward and Tn5-specific reverse primers. Amplicons are then subjected to NGS	Can detect and quantify SVs where the break point is proximal to the primer site. Can also be used to identify candidate off-target sites	Low or variable efficiency of DSB and tag process. Acts as a reporter method. Cannot be applied to bulk DNA that was edited using alternative methods	(Yu et al. 2022)

*ddPCR* digital droplet PCR, *LOH* loss of heterozygosity, *CN-LOH* copy-neutral LOH, *HMW* high molecular weight, *MDA* multiple displacement amplification, *M-MLA* moloney murine leukemia virus, *NGS* next-generation sequencing, *SNP* single-nucleotide polymorphism, *UMI* unique molecular identifier

### Cytogenetic analysis (FISH, aCGH, and SNP-analysis)

Fluorescence in situ hybridization (FISH) uses fluorescent DNA probes to label the presence (or absence) of complementary regions of a chromosome, which are viewed in interphase or metaphase cells. These probes can be designed to flank a locus of interest to identify a change in chromosome ploidy post-editing, with a standard resolution of 50 kb (Martin and Warburton 2015). Chromosome arm truncations can be visualized with FISH using probes complementary to the centromeric and telomeric regions of the target chromosome arm where loss of the telomeric probe is indicative of a truncation (Cullot et al. 2019). This method is valuable when analyzing aneuploid cell lines, where SVs could be masked by homologous chromosomes (Cullot et al. 2019).

Array comparative genomic hybridization (aCGH) enables localized or genome-wide screening of DNA copy-number imbalances based on the relative intensity of sample and control DNA fragments attached to an array of complimentary fluorescent probes (Cullot et al. 2019). aCGH can assay any locus that is represented on an array with a theoretical resolution up to 500 bp dependent on the frequency and size of the probes (Conrad et al. 2009). However, aCGH can only detect copy number changes and is unable to detect balanced chromosomal SVs (inversions, translocations, and CN-LOH) or the location of the copies. Furthermore, aCGH is intended to be used on heterozygous cells, so its application is limited to the analysis of clonal cell lines.

SVs can also be detected via analysis of the allelic ratios of native single-nucleotide polymorphisms (SNPs) (Alanis-Lobato et al. 2021; Leibowitz et al. 2021; Przewrocka et al. 2020; Simkin et al. 2022; Weisheit et al. 2020; Zuccaro et al. 2020). In edited cells, the loss of heterozygosity across multiple concurrent SNPs indicates a deletion, while a consistent 1:2 SNP ratio indicates a duplication. SNP genotyping assays may entail simple PCR and Sanger sequencing of known SNPs, or microarrays which encompass SNPs across the human genome. The LOH of SNPs can be tracked along a chromosome arm to determine the extent of large deletions or identify chromosomal truncations, where the LOH will extend to the telomere. However, SNP-analysis methods are also restricted to clonal cell lines and act as an indicator of SVs as they are not typically able to resolve the SV to base pair resolution.

### Quantitative genotyping PCR

SVs which prevent PCR amplification can be detected by allele copy number analysis by real-time quantitative PCR (qPCR) (Boutin et al. 2021). In qPCR, intercalating fluorescent DNA dyes or fluorescently labeled oligo-probes are used during PCR to produce fluorescent amplicons. The fluorescence intensity, which is proportional to the quantity of amplicon at any moment, is measured after each PCR cycle. The cycle number where the fluorescence becomes detectable above background is called the quantification cycle (Cq). For allele copy number analysis, equimolar amounts of DNA from control and edited cell clones are run in parallel so that the Cq cycles can be compared. Cell clones with large deletions will have higher Cq values due to reduced allele copy number, while those with duplications may have lower Cq values due to an increase in allele copy number. Recently, quantitative genotyping PCR (qgPCR) was developed (Simkin et al. 2022; Weisheit et al. 2020, 2021). qgPCR is a combination workflow where the qPCR primers are designed to match standard genotyping PCR primers, so that both the genotype and allele copy number can be determined. For example, in the case of a heterozygous deletion which prevents amplification of one allele, a standard genotyping assay may indicate a homozygous genotype, but the qgPCR will have a higher Cq value, indicative of a deletion. This enables the detection of SVs that would not be detected by standard genotyping PCR due to the loss of a primer site. However, qgPCR acts primarily as an indicator of SVs and is not able to resolve the SV to base pair resolution.

### Targeted amplicon sequencing, long-range sequencing technologies and IDM-seq

Standard amplicon sequencing enables the detection of edits that are contained entirely within the amplicon. Thus, the amplification of longer DNA fragments enables the detection of larger SVs, but is limited by the length of the amplification fragments that can be produced, and the requirement of the presence of both primer sites. Long-amplicon sequencing refers to the production and sequencing of large amplicons, typically 5–20 kb, which would enable the detection of kilobase-sized SVs.

Long-read sequencing technologies, such as Oxford Nanopore Technologies (ONT) or Pacific Biosciences (PacBio), provide alternative methods to observe SVs in long amplicons. For a more detailed description of these technologies including their pros and cons, we direct readers to a recent review (Logsdon et al. 2020). In brief, these technologies generate reads that are kilobases to megabases in length, so can cover entire amplicons and generate reads that are likely to contain unique SVs. In contrast, short-read sequencing methods may not capture or only partially capture SVs, making the mapping and analysis of pooled DNA challenging. Although long-read sequencing technologies generally have lower base-pair accuracy than Illumina sequencing, high accuracy is not essential for the visualization of large SVs. Moreover, PacBio sequencing can achieve high accuracy with sufficient sequencing passes of the same amplicon (Logsdon et al. 2020), and ONT sequencing accuracy is continually improving with new sequencing platforms and reagents (https://nanoporetech.com/accuracy). A benefit of higher sequencing accuracy is that it may facilitate the detection of both SVs and the accurate quantification of desired edits (such as HDR and INDELs) in a single assay (Bi et al. 2020). PacBio sequencing can achieve high base pair accuracy up to 15–20 kb, but its throughput is still limited and its cost per base sequenced is comparatively high among NGS technologies (Logsdon et al. 2020). Similarly, ONT technologies face similar limitations, although the exact extent may vary depending on the experimental setup.

While sequencing PCR amplicons can be used to identify the presence of SVs, the process of amplification and sequencing can introduce PCR and sequencing duplicates, hampering the accuracy of quantification. Individual DNA molecule sequencing (IDM-Seq) prevents this using unique molecular identifiers (UMIs) and either short- or long-range PCR to quantify the abundance of SVs over a target site (Bi et al. 2020). In IDM-seq, addition of a UMI is achieved by performing a round of primer extension using a single-stranded primer (containing a 10-12 nucleotide UMI sequence and 5′ universal primer sequence) that is specific to the locus of interest. Subsequent PCR amplification is then performed with a universal primer and a locus-specific reverse primer. Labeled amplicons can then be sequenced on NGS platforms. The addition of UMIs enables accurate quantification of allele frequencies from the bulk DNA, as each UMI group represents a single DNA molecule present at the initial labeling step (Bi et al. 2020).

### SV capture techniques; LAM-HTGTS, PEM-seq and CAST-seq

**Fig. 2:**
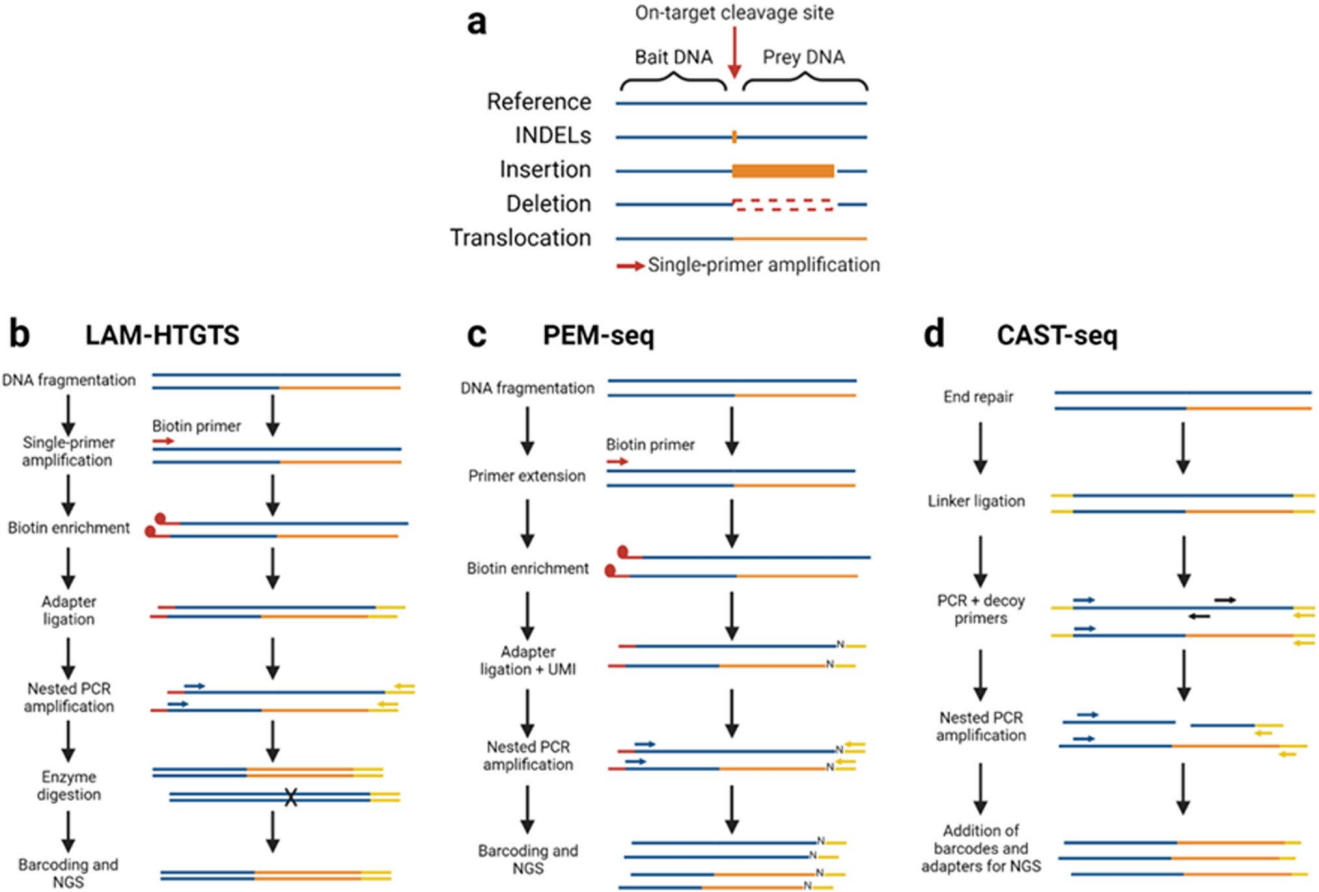
Diagrammatic overview of the bait/prey DNA system and SV capture techniques. **a** The bait DNA is the known sequence of DNA before the on-target cleavage site, which is followed by the prey DNA, the known sequence of DNA after the on-target cleavage site. Single-primer amplification from the bait DNA into the prey DNA can capture SV boundaries. **b**–**d** Schematic representations of the three SV capture techniques, LAM-HTGTS, PEM-seq, and CAST-seq which are described in detail in Sect. 3.4. Each diagram represents the workflow of the three techniques with one unedited allele (blue) and one allele with a translocation (blue/orange). The on-target cleavage site is located in the center of each amplicon (not depicted). Biotinylated primer sequences are shown in red, while linker and adapter sequences are in yellow. Primers are indicated by colored arrows and may be specific to the bait or prey DNA (blue, or red if biotinylated) or the linker/adapter sequence (yellow) **b** Enzymatic digestion of amplicons containing the unedited prey DNA sequence is indicated by the cross, ‘X’. **c** Unique molecular identifiers (UMI) which are added during adapter ligation are indicated by the ‘N’ on each amplicon. **d** ‘Decoy primers’ (black) are specific to the amplicons which contain the unedited prey sequence

SV capture techniques use linker-mediated amplification or single-primer PCR amplification across an on-target cleavage site to produce amplicons which may contain a SV boundary. These amplicons consist of a known sequence of DNA (bait DNA), followed by the prey DNA, which is either the reference or an aberrant DNA sequence (Fig. 2a). The nature of a SV can be resolved by mapping the prey DNA sequence to a reference genome. For example, in the case of a 5 kb deletion, the prey sequence will align to the reference genome 5 kb downstream of the bait sequence. If a translocation has occurred, the bait and prey DNA sequences will align to different chromosomes.

Several single-primer PCR amplification techniques have been developed, including linear amplification-mediated high-throughput genome-wide translocation sequencing (LAM-HTGTS) and primer extension-mediated sequencing (PEM-seq). LAM-HTGTS was developed to track translocations for the identification of off-target DSB sites. First, linear extension is performed with a biotinylated primer for ~ 80 cycles, followed by streptavidin-based isolation of amplicons (Fig. 2b) (Hu et al. 2016). Adapters are then ligated to the 3′ end of single-stranded amplicons and a nested PCR is performed using the adapter and a target-specific primer. In LAM-HTGTS, to improve the detection of variants, DNA molecules, which do not contain SVs, can be ablated via restriction digest using a rare-cutting restriction enzyme which cleaves unedited prey DNA. This step can be omitted in order to detect small repair events such as INDELs, and to allow quantification of the portion of fragments containing a SV. However, due to amplification and sequencing duplicates, the accuracy of quantification with LAM-HTGTS is limited.

PEM-seq is similar to LAM-HTGTS, but is able to accurately quantify allele frequency in pooled DNA due to the addition of UMIs before PCR amplification (Fig. 2c) (Yin et al. 2019). In PEM-seq, primer extension is conducted with a biotinylated primer for only one cycle, and amplicons are purified using streptavidin magnetic beads. Sequencing adapters with UMIs are then ligated onto the purified amplicons before a nested PCR, followed by NGS. This addition of a UMI means that each DNA molecule from the original pool of DNA before amplification is represented by a single UMI. This UMI can be utilized during analysis to accurately quantify variant frequency (both SV and INDELs) with high sensitivity (dependent on sequencing depth).

Chromosomal aberrations analysis by single targeted linker-mediated PCR sequencing (CAST-seq) is a method which can enrich and detect SVs using the bait/prey DNA system (Fig. 2d) (Turchiano et al. 2021). In CAST-seq bulk DNA is fragmented and then a linker is ligated to both ends. A subsequent PCR is performed using bait sequence specific and linker specific primers. ‘Decoy’ primers are also included which are specific to the reference genome within the prey sequence. Due to the presence of decoy primers, DNA fragments which retain the native prey DNA generate fragmented PCR products, rather than the full-length PCR products produced by those containing a SV. A second nested PCR is then performed using separate primers specific to the bait and linker DNA within the amplicons, allowing for amplification of fragments containing the locus of interest and a SV. A third PCR is performed to introduce a NGS barcode and adapter for sequencing. The CAST-seq method significantly enriches for SV containing sequences and has a detection threshold down to one SV per 10,000 cells. Furthermore, while the frequency of SVs in bulk DNA cannot be readily quantified, this can be achieved via a ddPCR calibration step using the same pooled DNA.

The benefits of using single-primer amplification for analysis of a targeted-DSB site are as follows: first, single-primer and linker-mediated PCR relies on only one loci-specific PCR primer for the initial amplification. Thus, they can detect SVs that cannot be detected by standard amplicon sequencing due to the removal of one primer binding site; second, the target primer can be placed either side of the target loci, which improves the detection capabilities; third, the amplification products are typically small, so can be robustly interrogated by NGS, even with pooled DNA. Finally, these techniques can identify SV junction points, including translocations, and if frequent these junctions may highlight off-target loci for further analysis.

While LAM-HTGTS, PEM-seq, and CAST-seq are useful for detecting SVs, these techniques have limitations associated with the use of PCR amplification. First, these rely on effective primer design, which may not be possible at all loci. Second, SVs where the specific (bait) primer site has been lost cannot be detected, as may be the case with large deletions. As these primers are typically located in close proximity to the target site (~ 200 bp), these techniques are best suited for the detection of insertions, inversions, translocations, and small deletions. However, large deletions which retain the specific (bait) primer site will be detected. While using a primer specific to a more distal site could improve the detection of larger deletions, this is limited by both the maximum size of amplicon that can be produced by PCR and the read length of the sequencing technique used. Finally, it should be noted that a ‘universal bait DSB’ strategy was introduced for LAM-HTGTS (Hu et al. 2016). This strategy may also be useful for PEM-seq and CAST-seq, eliminating the need for primer design at each targeted loci (more information can be found in the original paper) (Hu et al. 2016). However, the ‘universal bait DSB’ method is limited to the detection of chromosomal translocations.

### Xdrop

Xdrop™ is an indirect sequence capture system that circumvents the requirement for targeted PCR amplification over the break site in order to capture SV sequences (Blondal et al. 2021; Madsen et al. 2020). Xdrop capture is achieved by encapsulating DNA fragments of up to 100 kb into double emulsion droplets (water/oil/water) along with PCR primers that amplify a 100–200 bp product over 5 kb distal to loci of interest. PCR is then performed on the encapsulated DNA, followed by staining with a DNA-intercalating fluorescent dye and flow-assisted sorting to isolate fluorescent droplets containing the region of interest. The sorted DNA is then amplified by multiple displacement amplification (MDA) which amplifies large DNA fragments without requiring specified primer sets (Lasken 2009). The amplified enriched DNA is then prepared for sequencing by NGS. A key advantage of the Xdrop method is that it enables the examination of longer DNA amplicons (restricted by the average length of DNA) than could be possible by traditional PCR. In addition, the distance of the selection PCR primers from the target site means that they are not restricted in their design and are less likely to be removed by large deletions.

### PEAC-seq

Prime editor-assisted off-target characterization (PEAC-seq) is a technique that can detect off-target sites and translocations (Yu et al. 2022). It uses a Cas9 nuclease which is fused to the Moloney Murine Leukemia Virus (M-MLV) reverse transcriptase (RT) protein. The Cas9 creates a DSB at both on-target and off-target sites guided by the prime editing gRNA (pegRNA) sequence. The RT then introduces a "tag" sequence at the DSB site through reverse transcription of the RT template. Bulk edited DNA is fragmented using tagmentation with the Tn5 transposase, which incorporates adapter and UMI sequences to account for potential PCR and sequencing bias. Two separate PCR reactions are conducted to amplify both sides of the target site, each using a PCR primer that binds to the Tn5 and tag regions in either the forward or reverse direction. A subsequent PCR reaction is conducted on the products to add adapters for Illumina sequencing. Off-target sites are then identified by aligning the sequences surrounding the tag DNA to a reference genome. PEAC-seq can also be used to identify translocations at a known target site by substituting the tag specific primers with a site-specific forward primer. This reaction produces an amplicon that spans the target site into the candidate off-target region and can detect translocations from the target DNA with or without the tag sequence. PEAC-seq is however limited by the insertional efficiency of the PEAC-seq tag, which the authors note may vary between pegRNA and target loci.

### Alternative SV detection methods (Strand-seq)

While this review focuses on SV detection methods that have already been used to evaluate CRISPR-editing, other technologies that could aid in SV detection have not yet been incorporated into a CRISPR analysis workflow (Mahmoud et al. 2019). For example, Strand-seq is a single-cell sequencing method initially developed to track sister-chromatid exchanges (Falconer et al. 2012) but has also been utilized to detect other SVs such as deletions, duplications, inversions, and translocations (Jeong et al. 2022; Sanders et al. 2019). Further details about Strand-seq can be found in the primary sources (Falconer et al. 2012; Jeong et al. 2022; Sanders et al. 2017, 2019). Briefly, by sequencing single-chromosome strands, Strand-seq enables the differentiation of sequences between parental chromosomes, enabling a more robust evaluation of SVs than is provided by other single-cell and whole genome sequencing approaches. If utilized on a group of CRISPR-edited cells, Strand-seq has the potential to detect SVs across the entire genome, with the threshold for detection depending on the number of cells sequenced.

### Clonal expansion assays

Another method to measure the impact of CRISPR-induced SVs is by tracking the frequency of SVs over time or by performing assays to track the clonal expansion of edited cells. The SV detection methods mentioned above can be performed at subsequent time points during cell expansion to monitor the SV containing population of edited cells and to potentially identify the expansion of undesirable edits. However, this analysis will be limited to the types of SVs each method is able to detect. Alternative techniques are available to track the clonal expansion of edited cells within a heterogeneous population, although these are not necessarily specific for the detection of SVs (Sharma et al. 2021). One example is the TRACE-Seq method, which enables the introduction of the desired edit while also tracking the contribution of alleles and allele lineages (Sharma et al. 2021). This method involves generation of adeno-associated virus (AAV) libraries that have semi-randomized, silent mutations within the donor template, while also preserving the reading frame and capacity to induce the desired edit. Consequently, a pool of corrected cells with a diverse range of silent mutations is generated. The allelic contribution of the edited cells can be tracked by sequencing the target site using next-generation sequencing. If one allele increases in frequency or there is a significant change in allele contribution, it is indicative of clonal expansion of the cell containing those edits. Although TRACE-Seq is designed for AAV vectors, the same principles may be applied to any HDR approach by adding semi-randomized silent mutations to the donor template.

## Conclusion

Ex vivo CRISPR-Cas9 gene therapies have already advanced into stage 2 and 3 clinical trials for a number of genetic diseases (Chen et al. 2021). In vivo gene therapies, which deliver CRISPR machinery directly into the body via adenoviral or lipid nanoparticle vectors, have also recently moved into human trials (Taha et al. 2022). Although unanticipated genotoxic events in the form of small INDELs at off-target sites are routinely evaluated, new data suggests that large on-target SVs are also consequential editing outcomes that require their own evaluation. This can be technically challenging given their diverse and complex nature. While most analysis methods have a relatively limited capacity to detect various SV classes, others, such as PEM-seq, CAST-seq and Xdrop can detect many SV types from bulk edited genomic DNA. Henceforth, it will be important to combine multiple modes of analysis to ensure the maximum detection of both small INDELs and large SVs.

To date, as described above, large deletions, insertions, inversions, rearrangements, chromosomal truncations, CN-LOH, translocations, and chromothripsis have all been described in various CRISPR-edited primary human cells and human cell lines. None of these would have been detected by “standard” genotyping analysis methods. Interestingly, themes regarding the dominant class of SV in each cell type have begun to emerge. As may be expected, aneuploid cancer cell lines with natural chromosomal instability are more prone to large chromosomal aberrations, such as truncations and translocations, compared to karyotypically stable cell lines (Rayner et al. 2019). This is likely due to a difference in regulation of key DNA repair and checkpoint proteins, such as the tumor suppressor protein p53. In genetically stable cells, moderate kilobase-sized deletions, insertions and rearrangements seem to be the prominent on-target SVs (Turchiano et al. 2021). However, CN-LOH of entire chromosome arms and low-level translocations have also been detected (Boutin et al. 2021; Leibowitz et al. 2021). The predisposition for “small”, or copy number neutral aberrations in primary cells is likely explained by the negative selection pressure of large genomic imbalances at cell cycle checkpoints (Mirgayazova et al. 2020). So, although chromosomal aberrations may be less frequent in genetically stable cells, long-term studies which track the frequency of SVs are warranted.

If not properly accounted for, on-target SVs may deleteriously impact the validity and safety of CRISPR-Cas9 research. There are now substantial precedents which indicate that standard short-amplicon analysis methods do not detect most SVs which may have significant downstream functional consequences (Boutin et al. 2021; Weisheit et al. 2020). Fortunately, as far as we are aware, no adverse events have occurred due to unintended on- or off-target CRISPR-editing in clinical trials to date. Nonetheless, it may be prudent to proceed with caution until the prevalence and the impact of large genomic aberrations are better understood. The newest generation of CRISPR-Cas tools has the potential to completely avoid DSBs and, hopefully, their associated genotoxic effects (Anzalone et al. 2020; Cullot et al. 2019; Yin et al. 2019). However, even with the development of these tools, a comprehensive understanding of all editing outcomes, from small INDELS to SVs will only serve to improve the safety of CRISPR-Cas therapies.

Finally, the aim of this review is to assist researchers who may be using the CRISPR system in a diverse range of applications. Since each application carries a varying risk of both generating SVs and risk incurred from SVs, it is the responsibility of the researcher to determine the level of concern on a case by case basis. We recommend that researchers attempt to quantify SVs when reporting editing efficacy from bulk edited cells and to determine allele copy number if working with clonal cells. This is especially important when working with cancer cells where cytogenic analysis may also be performed. In addition, functional assays could be included alongside genome editing results wherever feasible. CRISPR-based therapies and their gene targets are varied. Therefore, prior to any clinical trial, it is crucial that they undergo rigorous preclinical testing and that this process includes a robust analysis of SVs.

## Data Availability

Data sharing is not applicable to this article as no new data were created or analyzed in this study.
